# Risk-management decision-making data from a community-based sample of racially diverse women at high risk of breast cancer: rationale, methods, and sample characteristics of the *Daughter Sister Mother Project* survey

**DOI:** 10.1186/s13058-023-01753-x

**Published:** 2024-01-11

**Authors:** Tasleem J. Padamsee, Christina Bijou, Paige Swinehart-Hord, Megan Hils, Anna Muraveva, Rachel J. Meadows, Kate Shane-Carson, Lisa D. Yee, Celia E. Wills, Electra D. Paskett

**Affiliations:** 1 Division of Health Services Management and Policy, College of Public Health, The Ohio State University, 280F Cunz Hall, 1841 Neil Avenue, Columbus, OH 43220 USA; 2 Department of Sociology, The Ohio State University, Columbus, OH USA; 3 Government Resources Center, The Ohio State University, Columbus, OH USA; 4 Center for Epidemiology and Healthcare Delivery Research, JPS Health Network, Fort Worth, TX USA; 5 College of Medicine, The Ohio State University, Columbus, OH USA; 6 City of Hope Comprehensive Cancer Center, Duarte, CA USA; 7 College of Nursing, The Ohio State University, Columbus, OH USA; 8 James Comprehensive Cancer Center, The Ohio State University, Columbus, OH USA

**Keywords:** Breast cancer prevention, Breast cancer risk, Survey methodology, Health disparities, Cancer health disparities, Community-based sample, Breast cancer risk management

## Abstract

**Background:**

To understand the dynamics that limit use of risk-management options by women at high risk of breast cancer, there is a critical need for research that focuses on patient perspectives. Prior research has left important gaps: exclusion of high-risk women not in risk-related clinical care, exclusion of non-white populations, and lack of attention to the decision-making processes that underlie risk-management choices. Our objective was to create a more inclusive dataset to facilitate research to address disparities related to decision making for breast cancer risk management.

**Methods:**

The *Daughter Sister Mother Project* survey collects comprehensive information about the experiences of women at high risk of breast cancer. We collected novel measures of feelings about and reactions to cancer screenings; knowledge, barriers, and facilitators of risk-management options; beliefs related to cancer risk and risk management; and involvement with loved ones who had cancer. Eligible individuals were non-Hispanic white and non-Hispanic Black adult women who self-identified as having high risk of breast cancer and had no personal history of cancer. Between October 2018 and August 2019, 1053 respondents completed the online survey. Of these, 717 were confirmed through risk prediction modeling to have a lifetime breast cancer risk of ≥ 20%. Sociodemographic characteristics of this sample were compared to those of nationally representative samples of the US population: the 2019 Health Information National Trends Survey and the Pew Research Center report: *Jewish Americans in 2020*.

**Results:**

The sample of 717 women at objectively high risk of breast cancer was largely (95%) recruited from non-clinical sources. Of these respondents, only 31% had seen a genetic counselor, 34% had had genetic testing specific to breast cancer risk, and 35% had seen at least one breast or cancer care specialist. The sample includes 35% Black respondents and 8% with Ashkenazi Jewish ancestry. Although encompassing a substantial range of ages, incomes, and education levels, respondents are overall somewhat younger, higher-income, and more educated than the US population as a whole.

**Conclusions:**

The DSM dataset offers comprehensive data from a community-based, diverse sample of women at high risk of breast cancer. The dataset includes substantial proportions of Black and Ashkenazi Jewish women and women who are not already in clinical care related to their breast cancer risk. This sample will facilitate future studies of risk-management behaviors among women who are and are not receiving high-risk care, and of variations in risk-management experiences across race and ethnicity.

## Background

In the USA, women at high risk of breast cancer face a 20–72% lifetime risk of the disease—much higher than the average 13% in the general population—due to known pathogenic genetic variants or strong family histories of breast, ovarian, and other related cancers [1, 2]. A range of clinical options exist to assist such women and use of these methods is essential to both individual and population health. At the individual level, enhanced surveillance methods, including annual screening breast magnetic resonance imaging (MRIs) as well as initiation of annual screening mammograms at an earlier age, can substantially improve the chances of detecting breast cancer early—when treatment is most effective. In addition, prophylactic surgeries (i.e., bilateral mastectomy and oophorectomy) and preventive medication can reduce breast cancer risk by 40–95% [3, 4]. The proportion of women at high risk of breast cancer in the general population has not been ascertained; studies of genetic risk focus solely on cancer patients or on the prevalence of specific pathogenic variants. We do know that the prevalence of inherited pathogenic *BRCA* variants in the general population is approximately 0.2–0.3%, that about 2.0% of people of Ashkenazi Jewish descent carry such variants, and that the overall population proportions at high risk are substantially higher than these figures—perhaps up to 15% [2, 5]. At the population level, 5–10% of breast cancers are hereditary, and the available risk-reduction methods could potentially eliminate the majority of morbidity, mortality, and associated financial costs due to these cancers [6]. Although the potential benefits are substantial, all risk-reduction options are substantially underused by the patients for whom they are recommended [7–10]. There is a critical need for research that focuses on patient perspectives to help understand psychological, interpersonal, social, and structural barriers to use of risk-reduction options among high-risk women. Such information is also critical to the development of health and social interventions that may improve the use of risk-reduction options among diverse groups of high-risk women.

Quantitative research over the past two decades has examined important patterns in decisions about a range of risk-management options but has also left three critical gaps that result from existing dataset limitations. First, most prior studies have been based on samples drawn from specific clinical groups—often from a single clinical site—instead of broader populations of high-risk women. Many studies have explored genetic testing and preventive surgery choices among women with pathogenic *BRCA* mutations [11–27]; several additional studies have focused on risk-management among genetic counseling patients [28–35] as well as women who are already in clinical care related to elevated breast cancer risk [36–46]. Although these clinical samples have allowed researchers to illuminate key correlates of risk-management decisions and suggest actionable targets for intervention, they have left open a range of questions about risk-related knowledge, emotions, coping, and decision making among the potentially large proportion of high-risk women who are not in clinical care related to breast cancer risk [7, 9, 10]. A few studies have created non-clinical samples to help address these questions, recruiting respondents from a non-profit organization serving high-risk women [47, 48] or advertisements to attract high-risk women [49]. Others have sampled from a registry of family members of breast cancer patients [50], a full regional population of Jewish women [51], or high-risk women identified by abstracting medical records of patients served by large healthcare systems [52–59]. Large datasets designed to facilitate the study of risk-management decision making among all high-risk women—and not only those already in clinical care—remain a rarity.

A second omission of most existing studies of high-risk decision making is any examination of racial variation or disparities, which copious evidence demonstrates are evident in nearly every area of healthcare access and health outcome across the USA and thus specifically relevant for US women at high risk of breast cancer [60–62]. This problem originates in study design and the resulting limitations of datasets on which risk-management studies are based. The US population is just over 23% non-white [63], and nearly all existing samples substantially under-represent non-white populations. USA-based studies generally use samples that include at least 90% white respondents [11, 12, 29, 34, 37–40, 44, 47, 52–55, 58]; a few include between 10 and 20% non-white respondents [28, 35, 36, 48], and some do not report racial distribution of respondents at all [13, 23, 25, 27, 44–46, 49, 50]. One small pilot study included 30% non-white respondents [55], and a single large study included 34% non-white respondents [58]—these investigations are the first to base their analyses on samples that open the door to the critical examination of racial variation and disparities related to risk-management decision making in the USA.

Finally, most existing studies take a very narrow approach to risk-management decision making [7]. Some of the research in this area focuses only on the risk-management decisions that are made, without any attention to the underlying factors that influence decisions [11, 20, 23, 24, 26, 27, 36, 41, 42, 54, 57, 64]. Many studies include a small number of correlates that are associated with individual risk-management choices—these correlates most often include knowledge, risk perceptions, and cancer-related distress, anxiety, or worry [13, 17, 30, 34, 35, 40, 44, 46, 49, 51–53, 56, 65]. Research on specific interventions or decision aids often explores the specific decision-related reasoning those interventions are meant to assist [13, 25, 45, 55, 59]. A few studies have explored patients’ explicitly stated reasons to use or not use risk-management options [15, 18, 29, 38, 47, 58], as well as beliefs, values, information-seeking, communication, or interpersonal influences [28, 39, 43, 48, 50]. While investigating a single risk-management decision or a focused set of psychological decision correlates makes sense for individual studies, such studies do not aggregate to produce a comprehensive examination of the experiences and challenges high-risk women face when learning and deciding about risk-management options. Moreover, learning and decision making about many different options often occurs during the same time period. Only a small number of quantitative studies have begun to approach risk-management decision making as a more complex process, incorporating a broader range of predictive factors, mechanisms, or risk-management outcomes [12, 22, 37].

The *Daughter Sister Mother Project* (DSM) team, based at the Ohio State University (OSU), designed a novel survey instrument to study the experiences of high-risk women, and collected data from over 1,000 Black and non-Hispanic white women about their experiences, feelings, and choices related to their elevated risk. Our core objectives were to assemble a new dataset that: (1) is primarily *community-based* instead of clinically-based—to better represent the broad population of high-risk women, many of whom are not already in clinical care related to their breast cancer risk, (2) is *racially diverse*—to facilitate insight into disparities related to breast cancer prevention among high-risk women, and (3) documents *decision-making processes*—to facilitate deeper study of the determinants of the full scope of risk-management behavior than is possible using data focused solely on single risk-related decisions, individual health-related outcomes, and narrow lists of psychological correlates.

The purpose of this article is to describe the unique resulting dataset, including (a) our methods of survey development, recruitment, and risk prediction modeling from self-reported data, and (b) the sociodemographic characteristics of this initial sample from the DSM project, and how they compare with nationally representative samples of the US female population. Our research establishes the feasibility of collecting patient-centered perspectives from a community-based, racially diverse sample of high-risk women. The resulting DSM dataset offers the opportunity to study a wide range of research questions pertaining to risk-related decisions and experiences.

## Methods

### Survey development

To ensure a comprehensive and accurate understanding of the concepts for which survey data about the experiences and decisions of diverse high-risk women should be collected, the DSM team began with a qualitative, inductive study of high-risk women’s information gathering, coping with risk, and risk-relevant decision-making processes. In-depth interview data were collected from 50 Black and non-Hispanic white women recruited through the ResearchMatch and Study Search databases for research volunteers (see “Respondents, Recruitment, and Data Collection” section for details about these databases), from among patients of clinical genetics and high-risk breast programs at the OSU Comprehensive Cancer Center (OSUCCC), and by snowball sampling from interviewed informants. These interviews shed new light on the range of facilitators and barriers that influence risk-management decision making, and the ways these dynamics may vary across race, socioeconomic status, and other social characteristics. Among the novel findings from this study were (a) the ways that risk-management decisions are affected by the quality of patients’ relationships with their primary care providers and their ability to access detailed risk-related information from genetic counselors, oncologists, or breast specialists, (b) the importance of social support specific to the context of breast cancer risk and the impact of close exposure to cancer among loved ones on women’s coping with risk, and (c) the staged process of decision making about preventive mastectomy, preventive oophorectomy, chemoprevention, and enhanced screening routines. Results from this qualitative research have been published elsewhere [8–10, 66–68].

Findings from the DSM qualitative study, as well as from prior studies published by other research teams such as those cited above, formed the foundation for the conceptual outline of the DSM survey content. Where possible, the DSM survey utilized validated measures of our concepts of interest; in some cases, these were modified for use in the study of high-risk women (see examples in Table 1). Where such measures did not exist, we developed novel measures designed to operationalize concepts illuminated by the qualitative research. These concepts included: feelings about and reactions to cancer screenings; knowledge of risk-management options and reasons to use or not use them; beliefs related to cancer risk and risk management; and types of involvement with loved ones who had cancer. New measures were pre-tested and revised through a series of six cognitive interviews with eligible women [69]. The survey was programmed using the REDCap data collection system, available on any Internet-enabled device. Due to concerns about length, the final instrument was split into a main survey (designed to take 20–30 min to complete) and a supplemental survey to be offered for separate completion afterward (designed to take 10–15 min to complete). We confirmed technical function and length of the instrument by administering the main survey and supplemental surveys to 12 and 11 eligible women, respectively. Table 1 describes all the measures included in the DSM survey, along with their origins.

**Table 1:** Contents and origins of *Daughter Sister Mother Project* survey instrument items

Topic	Concepts measured	# Questions	Origin of measures
Daughter Sister Mother Project Main Survey		Total of 90–233 questions per respondent	
Personal information	Race, Hispanic ethnicity, Ashkenazi Jewish descent	2	Alliance^1^, newly developed questions adapted from IBISv7^2^, Claus^3^, and Gail^4^ risk prediction models
	Height, weight, age	3	Newly developed
	Biopsy history	1–3	Newly developed questions adapted from IBISv7, Claus, and Gail risk prediction models
Hormonal & reproductive history	Menstrual history	2–3	Newly developed questions adapted from IBISv7, Claus, and Gail risk prediction models
	Childbearing history	1–2	Newly developed questions adapted from IBISv7, Claus, and Gail risk prediction models
	Use of hormone replacement therapy	1–4	Newly developed questions adapted from IBISv7, Claus, and Gail risk prediction models
Risk perception	Perceived personal risk of breast cancer, cancers other than breast/ovarian	2	Gramling et al. (2006), Gurmankin Levy et al. (2006)
Cancer prevention beliefs	Belief that one can lower breast cancer risk	1	HINTS^5^
Family cancer history	History of cancer among relatives	9–30	Newly developed questions adapted from IBISv7, Claus, and Gail risk prediction models
Screening behaviors	Mammogram history and feelings	1–7	BRFSS^6^ & ACS^7^ (most recent, frequency)
			Newly developed (abnormal results, feelings, reasons not to be screened)
	Breast MRI history and feelings	1–7	BRFSS & ACS (most recent, frequency)
			Newly developed (abnormal results, feelings, reasons not to be screened)
	Clinical breast exam history and feelings	1–6	BRFSS & ACS (most recent, frequency)
			Newly developed (abnormal results, feelings, reasons not to be screened)
Genetic testing	Personal history of genetic testing, having heard of it, referral to genetic testing, stage of genetic testing decision, reasons for/against testing	2–6	Partially adapted from Watson et al. (1999), Green et al. (2009), newly developed
	Family genetic testing history	1–9	Newly developed questions adapted from IBISv7, Claus, and Gail risk prediction models
Preventive mastectomy	Personal history of preventive mastectomy, having heard of it, stage of decision making, difficulty of decision, reasons for/against it, conditions under which would consider it, barriers to surgery, breast reconstruction, relevant feelings	1–9	Newly developed
Preventive oophorectomy	Personal history of preventive oophorectomy, having heard of it, stage of decision making, difficulty of decision, reasons for/against it, conditions under which would consider it, barriers to surgery, relevant feelings	1–9	Newly developed
Chemoprevention	Personal chemoprevention history, having heard of it, stage of decision making, difficulty of decision, reasons for/against it, conditions under which would consider it, barriers to use, relevant feelings	1–13	Newly developed
Lifestyle behaviors	Tobacco smoking behavior	1–2	BRFSS
	Alcohol use	1–2	NHANES^8^
	Exercise habits	1	HINTS
	Fruit and vegetable consumption	1	Alliance (adapted form)
	Belief that lifestyle changes could lower risk	1–2	Newly developed
Cancer exposure	Number of cancer cases among loved ones, relationships involved, outcomes of cancer cases	1–4	Newly developed
	Emotional and physical exposure to cancer cases among loved ones	15–45	CONNECS Scale^9^, with the addition of a newly developed “direct involvement” subscale
Healthcare providers	Source of usual care	1–2	CITIES^10^
	Use of relevant healthcare providers—primary care providers and specialists	2	Newly developed
	Gender, length of relationship, quality of care, shared decision-making, referral patterns, discussing breast cancer risk w/ relevant providers	5–24	Newly developed, Ayanian et al. (2010), Control Preference Scale^11^ (adapted form)
Social support	Available types of social support	5	Medical Outcomes Study (MOS) Social Support Survey^12^ (adapted form)
	Supportiveness of family, friends, partner	3	Newly developed
Financial constraints	Current and past health insurance	2–4	CITIES, newly developed
Health status	Overall quality of life and health	2	CITIES
	General mental health	5	MHI-5^13^
	Cancer worry	3	Impact of Events Scale^14^ (adapted form)
	Comorbidities and limitations of daily living	2	Newly developed, partially based on Satariano and Ragland (1994)
Socioeconomic status, demographics	Household income and size, occupation, education, sexual orientation	5	CITIES, Alliance
Closing questions	Willingness to participate in future studies	2	Newly developed
	Providing email address for gift certificate	1	Newly developed
Daughter Sister Mother Project Supplemental Survey		Total of 37–45 questions per respondent	
Risk perception	Salience, severity belief, and fear of breast cancer	3	Gramling et al. (2006), Gurmankin Levy et al. (2006), newly developed items
Information gathering	Desire for information, importance of information, fear of information, confidence in obtaining information, barriers to obtaining information	5	Health Information Orientation Scale^15^, Autonomy Preference Index^16^, HINTS, Barriers to Information Scale^17^
Cancer exposure	Exposure to chemotherapy of a loved one	1	LILAC^18^
Social support	Experience & interest in support groups, experience and interest in other high-risk women, social support from partner	9–11	Newly developed, Watts et al. (2011)
Financial constraints	History of delaying/avoiding medical appointments/procedures due to financial constraints	7	CITIES (adapted form), NHIS^19^ (adapted form)
Caregiving responsibilities	Caregiving responsibilities for children or disabled/ill adults	2–5	Newly developed
Medical mistrust	Healthcare system mistrust	5	Health Care System Distrust Scale^20^
Discrimination	Experience of discrimination in health care	1–2	CARE^21^ (adapted form)
Religion & faith	Religious identification, attendance, strength of faith, perception that God controls health	4–6	CITIES, Schwartz et al. (2000), God Locus of Health Control scale^22^, newly developed

^1^Kahn et al. 2021 [102], Paskett, ED 2015: The Alliance Patient Questionnaire: A Pilot Study to Determine Questionnaire Feasibility (Alliance A191401)

^2^Tyrer, Duffy, and Cuzick 2004 [72]

^3^Claus, Risch, and Thompson 1994 [73]

^4^Gail 2015 [74]

^5^Health Information National Trends Survey (HINTS) https://hints.cancer.gov/ [76]

^6^Behavioral Risk Factor Surveillance System (BRFSS) https://www.cdc.gov/brfss/index.html [100]

^7^American Community Survey (ACS) - https://www.census.gov/programs-surveys/acs [99]

^8^National Health and Nutrition Examination Survey (NHANES) https://www.cdc.gov/nchs/nhanes/index.htm [86]

^9^Hawkins et al. 2012 [85]

^10^Community Initiative Towards Improving Equity and Health Status (CITIES)—Paskett et al. 2019 [98]

^11^Denger, Sloan, and Venkatesh 1997 [97]

^12^https://www.rand.org/health-care/surveys_tools/mos/social-support.html [96]

^13^Mental Health Inventory 5—Cuijpers et al. 2009 [95]

^14^Horowitz et al. 1979 [94]

^15^DuBenske et al. 2009 [93]

^16^Ende et al. 1989 [92]

^17^Gustafson et al. 2005 [91]

^18^Life and Longevity After Cancer (LILAC)—Paskett et al. 2018 [90]

^19^National Health Interview Survey (NHIS)—https://www.cdc.gov/nchs/nhis/index.htm [89]

^20^Shea et al. 2008 [88]

^21^Paskett et al. 2020 [101]

^22^Wallston et al. 1999 [87]

### Respondents, recruitment, and data collection

**Fig. 1:**
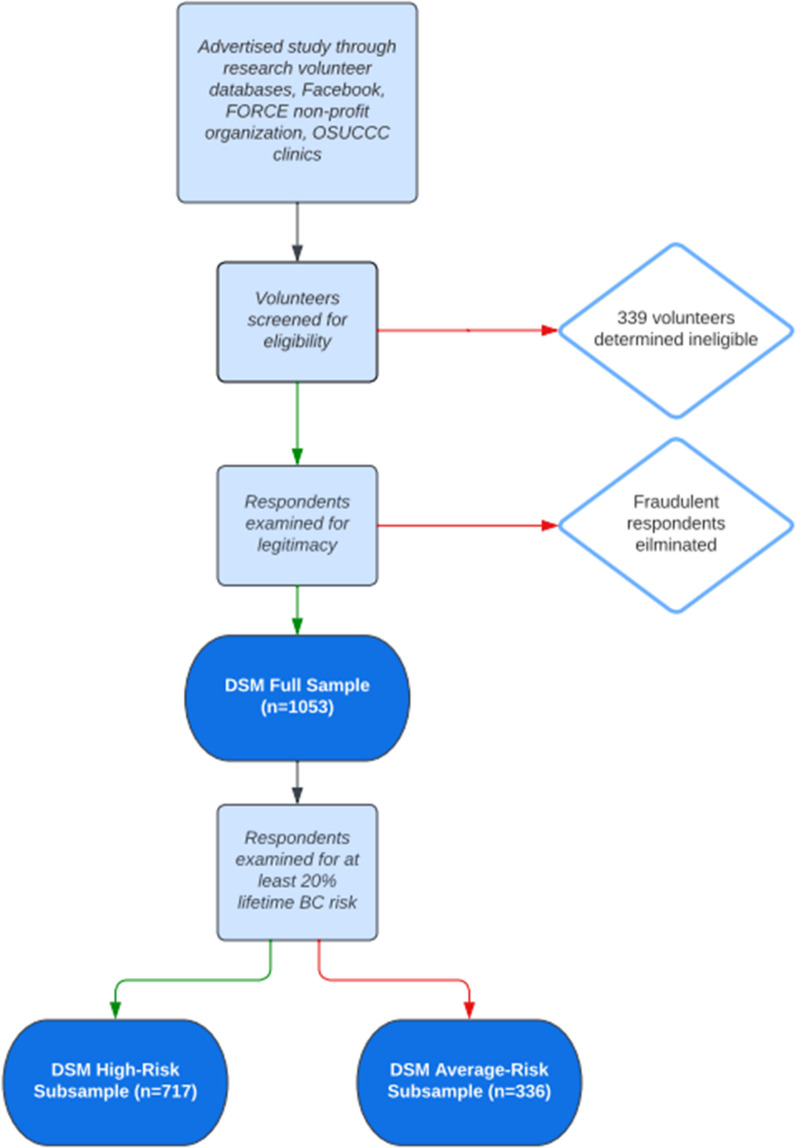
DSM survey recruitment and screening process

The recruitment and screening process is summarized in Fig. 1. DSM survey data were collected online between October 2018 and August 2019. Women eligible to participate were Black and non-Hispanic white women between 18 and 75 years of age, who had never had cancer (except non-melanoma skin cancer), who self-identified as having a potentially high risk of breast cancer (defined on study advertisements as having a family history of breast cancer or a *BRCA* mutation), and who were eligible after completing a screening survey. The screening instrument included up to 12 preliminary questions about gender, race, ethnicity, age, presence of family breast cancer history, and personal history of cancer, biopsies, and genetic testing. The DSM screening survey was used to determine whether potential respondents were likely to have an objectively high risk of breast cancer; 339 potential respondents screened out at this stage did not proceed to complete the main survey.

To include a large sample of high-risk women representing a more comprehensive range of high-risk women than usually studied, we recruited respondents from four sources: (1) two online databases that directly advertise academic studies to individuals who wish to volunteer as research participants: ResearchMatch (a national, non-profit, NIH-funded database that includes individuals who have signed up to indicate their interest in participating in medical research; anyone can sign up to be included) [99] and StudySearch (a listing of OSU-based studies that is searchable by all members of the public) [100]; (2) newsletters and other communications emailed to individuals involved with FORCE (Facing our Risk of Cancer Empowered, the largest US national non-profit organization providing information, support, and resources to individuals at hereditary risk of cancer) [70]; (3) Facebook advertisements tailored first to all women at high risk of breast cancer and then specifically to Black high-risk women; and (4) patients of clinical genetics and high-risk breast programs at the OSU Comprehensive Cancer Center (OSUCCC) and of select high-risk providers at other US clinical centers. Recruitment methods aimed to oversample Black women and to equalize—as much as possible—the number of respondents from each racial group generated from each recruitment source. Respondents were offered a $10 amazon.com gift card as incentive for completing the main survey; an additional $5 was offered for completing the supplemental survey.

The inclusion of online and social media advertising introduced a problem with fraudulent respondents (i.e., individuals completing the survey more than once and automated bots programmed to complete the survey repeatedly) that is commonly encountered in online surveys. We used a combination of methods to determine fraudulent responses; details of the full range of methods used to distinguish and eliminate data from fraudulent respondents will be described in a separate paper and is available on request [71]. Each survey we eliminated was determined to be fraudulent because: (1) it came from an individual we had not purposefully recruited from a volunteer database or patient list; (2) the data reflected at least one but usually multiple problems of these types: a mismatch in answers to questions about self-reported race or genetic testing that were asked on both the screening and the main survey, reporting of a very unlikely age (e.g.: first period or menopause at age 22), having had a biopsy without any prior breast screening or breast exams, completing the main survey in less than 20 min; and (3) when we called the phone number that had been provided for confirmation purposes we found that it was invalid, that the person who answered said no-one at the number had recently completed an OSU study, or—in a few cases—that no-one could be reached after multiple attempts. Because many fraudulent respondents started or completed the survey many times it is not possible to report the number of unique fraudulent respondents who accessed the survey; the number of individual surveys determined to be fraudulent was 1883. A total of 1,053 legitimate respondents completed the main survey—these respondents constitute our *DSM full sample*. Eighty-two percent (n = 866) of these individuals also completed the supplemental survey.

### Risk prediction modeling and eligibility

Collecting data from a community-based sample of high-risk women required the development of methods to estimate respondents’ objective breast cancer risk from self-reported data. The DSM survey accomplished this by integrating questions about personal health and medical history, as well as family cancer history; this section of the survey involved between 9 and 30 questions depending on the extensiveness and complexity of the respondent’s family history. This series of questions included all information necessary to run three commonly used risk prediction models: Gail, Claus, and IBIS (Tyrer-Cuzick) version 7 [72–74]; this approach of identifying all individuals who are at high risk according to at least one of multiple models mimics that used by many US genetic counseling and breast oncology practices. Members of the research team were trained by an experienced genetic counselor (co-author KSC) and then manually applied each appropriate risk prediction model for each respondent. We have reported the details of this risk prediction modeling process, as well as its implications for future research and clinical care of high-risk women, in a separate paper [75]. Respondents who were found to have 20% or greater lifetime risk by at least one risk prediction model met the common US clinical criteria to be considered ‘high risk’, and therefore constitute our *DSM high-risk* subsample (3). This high-risk subsample includes 717 respondents and is the core sample to be used in future DSM project studies of risk-management decision making among high-risk women. The remaining 336 respondents constitute our *DSM average-risk subsample*; our research team is undertaking further studies of this group of women who have a high perceived risk of breast cancer, but did not meet clinical criteria to be considered objectively as ‘high risk’ at the time of data collection. Overall, two-thirds of our full DSM sample indeed qualified as high risk after objective risk assessment. This relatively high level of concordance between subjective and objective risk is likely due to two reasons. First, we did not merely ask women if they were at high breast cancer risk, we specified that they may be eligible if they had a family history of breast cancer or a *BRCA* mutation. Second, we utilized a short screening survey based on simple objective criteria associated with high risk; this instrument was specifically intended to increase the likelihood that individuals who screened into the main DSM survey would have objectively high breast cancer risk.

### Descriptive analyses of sociodemographic characteristics

We describe the sociodemographic characteristics (race, Ashkenazi Jewish ancestry, age, education, and income) of our primary sample of interest, the DSM high-risk subsample. Ideally, our subsample would accurately represent the full population of Black and white high-risk women across the country. Since the characteristics of that population are largely unknown, however, we instead describe the characteristics of our dataset and how they compare to other datasets that accurately represent the female population of the USA. Specifically, we utilize the 2019 Health Information National Trends Survey (HINTS 5, Cycle 3) sample as our comparator for race, age, education, and income, and the Pew Research Center’s 2020 report on Jewish Americans as our comparator for Ashkenazi Jewish ancestry. There are several nationally representative health datasets that could reasonably have been chosen as a comparator for our DSM dataset; these datasets all contain largely similar demographic questions. We chose HINTS as the most appropriate comparator for most variables because it uses the same measure of household income as the DSM dataset. We considered household income important because it is the single most commonly used indicator of socioeconomic status and financial constraints on healthcare behavior and health outcomes. HINTS differs from other surveys—and from the US Census—because it asks respondents specifically about the combined annual income of all family members *living within the household*. This specification ensures that estimates do not include income from extended family members living outside the household, allowing for more precise measures of socioeconomic indicators such as household income and poverty status.

Population weights are applied to all HINTS and Pew estimates in accordance with their unique sampling designs to approximate population trends on a national level. HINTS uses a two-stage address-based stratified sampling design which randomly identifies respondents based on their mailing addresses [76]. Pew utilizes a similar stratified address-based sampling design, but focuses instead on areas with high concentrations of Jewish adults [77]. Pew does not provide unweighted numeric counts of the sample they use to produce their estimates of Jewish ancestry; therefore, all count columns for Jewish ancestry are marked "NA." All DSM subsample estimates are unweighted.

*Race.* The DSM sample includes only non-Hispanic white and Black women. Our direct comparisons also use only the Black and white respondents from the HINTS and Pew datasets. Tables 2 and 3 compare the demographic distributions of the entire DSM high-risk subsample to the entire HINTS and Pew female samples on race, Ashkenazi Jewish ancestry, age, education, and income. Tables 4 and 5 compare the demographic distributions of the high-risk DSM subsample to the race-specific HINTS and Pew subsamples of Black and white women.

**Table 2:** Demographic characteristics, full DSM high-risk subsample, and HINTS/Pew subsample

	DSM high-risk subsample (N = 717)		HINTS(N = 2040)/Pew subsample*	
	Unweighted N	Unweighted % (or Mean)	Unweighted N	Weighted % (or Mean)
Race^Ꞌ^				
White	466	64.99%	1,610	83.80%
Black	251	35.01%	430	16.20%
Ashkenazi Jewish Ancestry**				
No	621	86.61%	NA	98.82%
Yes	60	8.37%	NA	1.18%
Missing	36	5.02%	NA	0.00%
Age^Ꞌ^				
18–24 years	91	12.69%	54	8.96%
25–34 years	228	31.80%	203	12.30%
35–44 years	152	21.20%	246	14.70%
45–54 years	122	17.02%	313	22.00%
55 years and older	124	17.29%	1,201	41.20%
Missing	0	0.00%	23	0.88%
Mean age	–	39	–	51
Education^Ꞌ^				
Less than high school	3	0.42%	73	5.06%
High school graduate	28	3.91%	353	22.10%
Some college	152	21.20%	592	38.80%
Bachelor's degree	224	31.24%	585	20.10%
Post-baccalaureate degree	309	43.10%	423	13.40%
Missing	1	0.14%	14	0.48%

*Pew does not provide unweighted figures for ethnicity data

**Pew used as comparator dataset

^Ꞌ^HINTS used as comparator dataset

**Table 3:** Socioeconomic characteristics, full DSM high-risk subsample, and HINTS subsample

	DSM high-risk subsample (N = 717)		HINTS(N = 2040) Subsample	
	Unweighted N	Unweighted % (or mean/median)	Unweighted N	Weighted % (or mean/median)
Household income				
$0 to $9,999	16	2.23%	141	8.67%
$10,000 to $14,999	18	2.51%	113	5.07%
$15,000 to $19,999	23	3.21%	107	4.60%
$20,000 to $34,999	54	7.53%	279	12.10%
$35,000 to $49,999	78	10.88%	262	14.40%
$50,000 to $74,999	129	17.99%	383	17.80%
$75,000 to $99,999	91	12.69%	250	12.30%
$100,000 to $199,999	191	26.64%	381	19.40%
$200,000 or more	52	7.25%	120	5.34%
Missing	65	9.07%	4	0.34%
Mean household income	–	$50,000–74,999	–	$50,000–74,999
Median household income	–	$75,000–99,999	–	$50,000–74,999
Federal income relative to poverty (FIP)				
Income < 100% FPL	40	5.58%	217	16.10%
Income 100–400% FPL	265	36.96%	1011	46.80%
Income > 400% FPL	347	48.40%	808	36.70%
Missing	65	9.07%	4	0.34%

**Table 4:** Demographic characteristics by race, DSM high-risk subsample, and HINTS/Pew subsample

	White sample				Black sample			
	DSM high-risk subsample		HINTS/Pew subsample		DSM high-risk subsample		HINTS/Pew subsample	
	Unweighted N	Unweighted % (or mean)	Unweighted N	Weighted % (or mean)	Unweighted N	Unweighted % (or mean)	Unweighted N	Weighted % (or mean)
Ashkenazi Jewish Ancestry**								
No	383	82.19%	NA	98.98%	238	94.82%	NA	99.99%
Yes	57	12.23%	NA	1.02%	3	1.20%	NA	< 0.01%
Missing	26	5.58%	NA	0.00%	10	3.98%	NA	0.00%
Age^Ꞌ^								
18–24 years	61	13.09%	44	9.93%	30	11.95%	10	3.92%
25–34 years	141	30.26%	162	11.60%	87	34.66%	41	16.10%
35–44 years	89	19.10%	203	14.10%	63	25.10%	43	17.40%
45–54 years	81	17.38%	231	20.70%	41	16.33%	82	28.60%
55 years and older	94	20.17%	957	43.10%	30	11.95%	244	31.10%
Missing	0	0.00%	13	0.50%	0	0.00%	10	2.89%
Mean age	–	40	–	51	–	37	–	48
Education^Ꞌ^								
Less than high school	1	0.21%	45	3.73%	2	0.80%	28	11.90%
High school graduate	13	2.79%	270	21.30%	15	5.98%	83	26.20%
Some college	88	18.88%	450	39.10%	64	25.50%	142	36.90%
Bachelor’s degree	146	31.33%	469	21.00%	78	31.08%	116	15.60%
Post-baccalaureate degree	218	46.78%	365	14.30%	91	36.25%	58	8.93%
Missing	0	0.00%	11	0.50%	1	0.40%	3	0.42%
Sample size	466		1,610 (HINTS)		251		430 (HINTS)	

**Pew used as comparator dataset

^Ꞌ^HINTS used as comparator dataset

**Table 5:** Socioeconomic characteristics by race, DSM high-risk subsample, and HINTS subsample

	White sample				Black sample			
	DSM high-risk subsample		HINTS subsample		DSM high-risk subsample		HINTS subsample	
	Unweighted N	Unweighted % (or Mean)	Unweighted N	Weighted % (or Mean)	Unweighted N	Unweighted % (or Mean)	Unweighted N	Weighted % (or Mean)
Household income								
$0 to $9,999	6	1.29%	71	5.23%	10	3.98%	70	26.50%
$10,000 to $14,999	9	1.93%	67	4.14%	9	3.59%	46	9.87%
$15,000 to $19,999	11	2.36%	72	4.21%	12	4.78%	35	6.64%
$20,000 to $34,999	26	5.58%	222	12.50%	28	11.16%	57	10.50%
$35,000 to $49,999	36	7.73%	202	14.20%	42	16.73%	60	15.10%
$50,000 to $74,999	72	15.45%	311	18.20%	57	22.71%	72	15.70%
$75,000 to $99,999	68	14.59%	210	13.00%	23	9.16%	40	8.86%
$100,000 to $199,999	149	31.97%	340	22.00%	42	16.73%	41	5.90%
$200,000 or more	42	9.01%	112	6.24%	10	3.98%	8	0.68%
Missing	47	10.09%	3	0.36%	18	7.17%	1	0.25%
Mean household income	–	$75,000–99,999	–	$50,000–74,999	–	$50,000–74,999	–	$20,000–34,999
Median household income	–	$75,000–99,999	–	$50,000–74,999	–	$50,000–74,999	–	$20,000–34,999
Federal Income to relative to Poverty (FIP)								
Income < 100% FPL	13	2.79%	115	12.20%	27	10.76%	102	36.10%
Income 100–400% FPL	142	30.47%	782	46.40%	123	49.00%	229	49.00%
Income > 400% FPL	264	56.65%	710	41.00%	83	33.07%	98	14.60%
Missing	47	10.09%	3	0.36%	18	7.17%	1	0.25%
Sample Size	466		1,610 (HINTS)		251		430 (HINTS)	

*Ashkenazi Jewish ancestry.* We include Ashkenazi Jewish ancestry because it is associated with significantly increased risk of pathogenic *BRCA* variants and breast cancer. Many American Jewish communities are aware of this information and pay special attention to breast cancer family history and risk, so individual risk-related and risk-management experiences may be different among this ethnic population than others. National estimates of the prevalence of Ashkenazi Jewish ancestry are not available through HINTS, so we instead used alternate data sources to generate estimates to be compared to the DSM sample. Specifically, we utilized Pew Research Center estimates of the number of Black and white Ashkenazi Jewish adults in the USA and compared these numbers to American Community Survey (ACS) estimates of the total number of Black and white Americans to approximate the percent of Black and white Ashkenazi Jewish adults in the USA.

*Age.* Age was recoded from the specific age in years reported by respondents into five categories from all data sources: 18–24 years, 25–34 years, 35–44 years, 45–54 years, and 55 years and older.

*Education.* Education was recoded into five categories from all data sources: less than a high school degree, high school diploma or GED, some college, bachelor’s degree, and postbaccalaureate study.

*Income.* In addition to our direct measure of household income, we used questions about household income, household size, and the U.S. Department of Health and Human Services’ 2019 Federal Poverty Guidelines to construct a family income relative to poverty (FIP) variable from the DSM, HINTS, and Pew datasets. FIP was categorized into the following levels based on eligibility for insurance subsidies under the Patient Protection and Affordable Care Act: low income (household income < 100% of federal poverty level for respondent’s household size (FPL)), middle income (100–400% of FPL), and high income (> 400% of FPL). We categorized 90.9% of DSM respondents into one of these three categories.

### Analysis of missing data

Rates of item missing data were assessed for all 94 variables that reflect questions asked of all respondents. These questions were distributed across all sections of the survey instrument, appearing throughout early, middle, and end sections of the main survey and in the supplemental survey. Missing value counts included both cases where a respondent indicated “prefer not to answer” and cases where a respondent did not answer the question at all. “Don’t know” was a substantively meaningful answer for most questions in this survey; “Don’t know” answers were therefore not assessed as missing. The percentage of missing values was less than 2% for all variables, with the exception of household income, which was missing for 7.8% of respondents. Bivariate logistic regressions were used to check for non-randomness of these missing values by race and age, at alpha = 0.05. These analyses revealed no statistically significant relationship between missing income data and race. The odds of not providing an answer to the income question increased by 2.7% with every year of respondent’s age.

## Results

All descriptions of DSM results below refer specifically to the DSM high-risk subsample because it most directly addresses the original objectives of the DSM dataset; this subsample includes 717 respondents who were ascertained to be at objectively high (≥ 20% lifetime) risk of breast cancer.

### Sample description

**Fig. 2:**
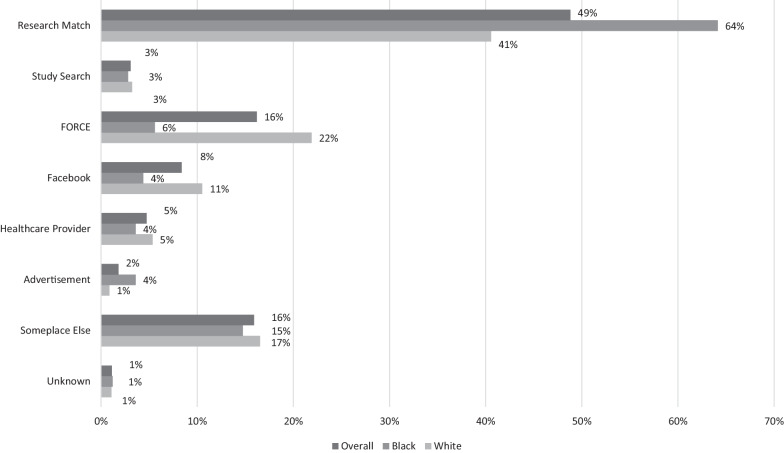
Recruitment sources for high-risk sample (n = 717), by race

Figure 2 presents the proportion of our 717 high-risk respondents who reported having accessed the survey from each of the seven recruitment sources and the racial distribution of respondents from each source. Just over half (52%) were recruited from one of the research volunteer databases—ResearchMatch or StudySearch, with most of these respondents resulting from our active efforts to recruit registered ResearchMatch users. Funded by the National Institutes of Health, ResearchMatch has 213 member institutions spanning the entire USA, which actively recruit and register willing research volunteers. Approximately 1 in 6 respondents (16%) was recruited through FORCE, a national non-profit organization that directly targets individuals at hereditary risk of cancer. Eight percent of respondents learned about the study via Facebook advertising. Overall, only 5% of the sample was recruited through a clinical source (i.e., a genetics or high-risk healthcare provider), and only 8% was recruited through a source embedded within the authors’ home institution. We characterize the sample as primarily community-based, since 95% of the sample was recruited from community-based sources as opposed to clinical ones. Research volunteer databases proved to be one of the most effective recruitment sources for Black respondents—nearly 67% of Black respondents were recruited from either ResearchMatch (64%) or StudySearch (3%), compared to 44% of white respondents. FORCE and Facebook were more effective among white than Black respondents, with almost 4 and 3 times as many white respondents being recruited from FORCE and Facebook, respectively.

### Race and ethnicity

Tables 2, 3, 4, and 5 present the sociodemographic characteristics of the high-risk subsample and compare them to other datasets designed to represent the overall US population. Our sample includes a considerably higher proportion of Black respondents (35%) than the HINTS sample (16.2%). Women of Ashkenazi Jewish descent comprise more than 12% of our white respondents, which is substantially higher than the general US population estimate of 1% produced by the Pew dataset.

### Age

Overall, DSM high-risk respondents are younger than HINTS respondents. Two thirds (65.7%) of DSM respondents are under the age of 45, compared to about one third (36.0%) of HINTS respondents. DSM respondents are also distributed more evenly across the life course than HINTS respondents, who are also more likely to be aged 55 or older (59.8% HINTS vs. 17.3% DSM). The age differences between DSM and HINTS samples are particularly pronounced among Black respondents: Only 37.4% of Black HINTS respondents are under 45 years, compared to 71.7% of Black DSM respondents. The average ages of Black and white DSM respondents are 37 years and 40 years, respectively; the 3-year difference is consistent with the HINTS dataset, in which the average ages of Black and white respondents are 48 years and 51 years, respectively (see Tables 2 and 4).

### Income

Household income in the DSM high-risk subsample is skewed high (median category = $75,000–99,999 per year) relative to estimates from the US Census 2019 (median = $68,703) and HINTS (median category = $50,000–74,999). In particular, the DSM dataset underrepresents households earning less than $50,000/year and over-represents households earning more than $100,000/year. Among HINTS respondents, 44.2% report household incomes under $50,000, in comparison to only 26.5% of DSM high-risk respondents. The modal income category for HINTS respondents is $50,000–$74,999, which is comparable to US Census estimates [78]; the modal income category for DSM high-risk respondents is $100,000–$149,999. The DSM sample resembles the HINTS sample more closely according to the family income relative to poverty (FIP) measure, although a higher proportion of DSM than HINTS respondents still fall in the high-income group (48.4% of DSM vs. 36.7% of HINTS) (see Table 3).

Differences in the income distributions of HINTS and DSM samples are larger when stratified by race. White HINTS respondents report lower household incomes than white DSM respondents—39.9% of white HINTS respondents vs. 18.9% of white DSM respondents report annual household incomes less than $50,000. In addition, 62.3% of Black HINTS respondents, but only 40.2% of Black DSM respondents, report annual household incomes below $50,000. Despite this, the modal income bracket is the same for DSM and HINTS respondents in each racial group. For white samples, this bracket is $100,000–$149,999 (32.0% for DSM and 21.1% for HINTS). For Black samples, the modal bracket is $50,000–$74,999 (22.7% for DSM and 16.7% for HINTS). Compared to white HINTS respondents on the FIP measure, more white DSM respondents fall into the high-income category and fewer white DSM respondents have low and middle incomes. More Black DSM than HINTS respondents are in the high-income group as well, while fewer Black DSM respondents are in the low-income group. The proportions of Black DSM and HINTS respondents in the middle-income category are equivalent (49%) (see Table 5).

### Education

Overall and within each racial group, the DSM sample is more educated than the HINTS sample. Specifically, the DSM sample contains triple the proportion of respondents with more than a college education (43.1% DSM vs. 13.4% HINTS) and very few respondents with less than a high school education (0.4% DSM vs. 5.1% HINTS). These patterns persist when the samples are stratified by race, although some become even more pronounced. For instance, 36.3% of Black DSM respondents had education beyond a bachelor’s degree, compared to 8.9% of Black HINTS respondents. Consistent with HINTS and other national data sources, our Black subsample is substantially less educated than our white subsample (see Tables 2 and 4).

### Use of risk-related care

The DSM sample was collected primarily from non-clinical sources in order to make it possible to assess the frequency with which a broader sample of high-risk women have accessed clinical care specifically related to their risk. Descriptive analysis of the DSM sample indicates that approximately a third of respondents had accessed such care: 31.2% had seen a genetic counselor, 34.5% had had genetic testing specific to breast cancer risk, and 35.2% had seen at least one breast or cancer care specialist.

## Discussion

Although women at high risk of breast cancer are relatively rare in the general population, it is critical that our research samples reflect the full population of these women as thoroughly as possible, and not be limited to particular demographic subgroups or women who have already accessed clinical high-risk care. Successful collection of the DSM dataset demonstrates the feasibility of generating comprehensive self-report data from a community-based, diverse sample of women at high risk of breast cancer. Because it is community-based, this sample offers new opportunities to study the risk-related attitudes and feelings, decision-making processes, and risk-management behaviors of women who know they are at high risk but may not have had the opportunity for clinical care related to that risk [9]. The racial and ethnic diversity of the sample is particularly unique and important. The DSM dataset includes responses from the highest proportion of Black respondents (35%) of any high-risk study we have identified from the literature and a substantial subsample (8%) of Ashkenazi Jewish respondents, which facilitates subgroup comparisons. The over-representation of Black women in the DSM dataset relative to the US population will facilitate studies designed to examine racial differences in high-risk and risk-management experiences. Our Ashkenazi Jewish subsample (n = 60) is large enough to facilitate analyses of the impact of this ethnic identity on experiences and decisions surrounding breast cancer risk.

The DSM dataset offers the opportunity to explore a wide range of previously unstudied questions about risk-management decision making among women at high risk of breast cancer. With the DSM high-risk subsample available, our research team is now pursuing important research objectives including the following; other teams may also utilize these rich data to pursue related objectives.

- Documenting how much—and what—a broad range of high risk women know about their own cancer risks and the full range of risk-reduction options. Risk and risk-reduction knowledge is a critical foundation for risk-management behavior, but gaps in this knowledge foundation cannot be fully examined using samples of women already in clinical care for their breast cancer risk.
- Determining the proportion of women who are in clinical care related to their breast cancer risk, as well as the predictors of and barriers to these avenues of clinical support. Specialist clinical care is necessary to access many forms of risk-management activity and also the most promising source for accurate risk and risk-reduction information.
- Understanding the experiences high-risk women have within high-risk clinical care, and potential avenues for improving the information, decision-making support, and access to risk-management options provided in those settings.
- Better documenting the gaps in women’s use of guideline-recommended risk-management methods by including women not already in clinical care, and understanding the causes of these gaps as a precursor to addressing them.
- Investigating how women’s attitudes, emotions, and beliefs contribute to their risk-management choices, and illuminating the interpersonal, social, and structural dynamics that also help drive those decisions. Understanding these drivers of risk management may suggest social, medical, and system changes that could better empower women to protect their future health.
- Exploring how all these patterns vary across race, the mechanisms of these associations, and the implications of these disparities for the health of Black populations. A solid understanding of disparities in risk-management decision making and behavior—or how to ameliorate them—is not possible without a diverse sample like the one provided by the DSM high-risk dataset.

The DSM average-risk subsample offers important research possibilities as well. This subsample opens the door to understanding the risk-related experiences of women who have a family history of breast cancer and therefore self-identify as possibly at elevated risk themselves, but who are not objectively at high (≥ 20%) lifetime risk. Among other topics, these data allow investigation of how lower-risk women with a family history understand their risk and cope emotionally, the degree to which they utilize regular screening methods, and how their risk-related thoughts, behaviors, and actions are similar to or different from women who have objectively higher risk and for whom specialized high-risk services are recommended.

While the DSM survey gathered important information about many aspects of risk-related decision making and will serve as a valuable tool for researchers studying these topics, collecting these data required coping with a range of challenges, and required considerable personnel and resources. One significant precursor to completing high-quality, rigorous analyses based on a mostly non-clinical sample of self-identified high-risk women was to ascertain an objective level of breast cancer risk for each respondent. We addressed this issue by collecting all the data required to manually run objective risk prediction models on each respondent—a method that maximizes the usefulness of self-report data, but also requires considerable research staff time [79]. An alternate approach would have been to incorporate screening phone calls into the recruitment process; a study staff member could have collected personal and family history and run risk prediction models before designating a respondent eligible to complete the main survey—this approach would reduce incentive payments to non-high-risk women but require multiple contact points with each respondent and move substantial staff effort to the screening phase. A third option would have been to automate the running of risk prediction models by linking self-reported personal and family history information to pre-programmed risk prediction models that are run online. At present, this approach would be feasible only for researchers who have the means to form contractual relationships with companies that can link their proprietary modeling software to self-reported data behind a confidentiality-protecting firewall. Novel programming of a system that could be made publicly available to future researchers would be a costly but valuable future innovation.

In order to maximize our opportunities to generate a large, diverse, community-based sample, we recruited from a variety of sources. Like other researchers, we found that social media advertising was highly effective but also introduced problems pertaining to fraudulent respondents, which were time-consuming and expensive to resolve [71]. Recruiting from databases of research volunteers was also a highly effective approach. ResearchMatch in particular is a large database that provides sufficient demographic information to allow targeting of recruitment efforts to locate specific types of respondents—this was helpful in recruiting a high proportion of Black women to the DSM sample. FORCE has proven to be an excellent source for women willing and motivated to participate in risk-related breast cancer research. With each of these recruitment sources in use, it proved possible to generate a large sample of respondents that was both racially diverse and not limited to patients of a particular clinical center.

Although the DSM dataset will allow a range of novel research questions to be asked and answered, it is not a fully representative sample of the entire population of high-risk women in the USA. In fact, the actual number and true demographic distribution of women at high risk of breast cancer in the United States are both unknown. All existing datasets have been ‘convenience’ samples of one type or another—drawn from cancer patients, their relatives, genetic counseling or testing patients, patients of high-risk clinics, or patients of particular local medical systems. While not determined by any of those specific constraints and broader than most prior samples, the DSM sample is also ultimately a ‘convenience’ sample, representing only groups of women who self-identify as at elevated risk of breast cancer, and who became aware of our study through the specific recruitment venues we used.

One specific omission of the DSM sample is women who are not aware of their high risk—who have neither become aware of this risk on their own nor been informed of it by a healthcare provider. This likely includes many women who theoretically *could* be identified as at high-risk, due to a known predisposing mutation in their family, their family history of breast and other related cancers, or other facets of their personal medical history. In this limitation, the DSM survey mimics all other survey-based samples derived from high-risk clinical settings or self-identification of breast cancer risk. Women who are unaware of their high risk are an important and neglected population, however, as they are particularly unlikely to have had access to risk-management methods that could protect their future health. In addition, although we specifically aimed to generate a more racially diverse sample than others available in order to facilitate disparity-related studies, our sample contains only white and Black women. This is a result of resource limitations, which did not allow for data collection of sufficiently large samples for analysis from more than two racial groups. Future samples should include other major US racial–ethnic groups, most importantly Hispanics/Latinas and Asian-Americans—the fastest growing US racial–ethnic groups, which also have increasing breast cancer incidence. Relatedly, future samples should also collect more nuanced information about respondents’ racial–ethnic origins; this would enable distinctions, for instance, between African-American and Caribbean Black populations.

To fully understand the size and demographic distribution of the high-risk population, and to consider interventions that could assist all groups in that population, it would be necessary pursue some form of population-based research. One approach might involve a comprehensive set of regional, population-based programs that identify all high-risk individuals through screenings of primary care patients, mammogram patients, patients of community-based and safety-net clinics, and/or patients of large healthcare systems. A related approach would involve recruitment through regional registries or mammography patients or cancer patients and their family members. Another approach would be to embed personal and family medical history questions (necessary to run risk prediction models) in a population-representative survey of the entire US population. This survey would also need to have sufficient sample size to locate an analyzable set of high-risk women, even though high-risk individuals are a small minority of the US population. Any comprehensive method of collecting data from the entire high-risk population would need to take particular care to include women with limited access to healthcare.

Despite its strengths, the DSM dataset also has specific limitations relevant to the interpretation of analyses. The data are cross sectional, retrospective, and self-reported; this will limit the ability to understand decision-making dynamics as they evolve over time and may introduce recall and reporting biases. Collecting data longitudinally and using medical records to confirm patient reports would appropriately fill these gaps. The DSM sample also includes only two of the five major US racial–ethnic groups; future studies would be improved by including Asian American, Latina, and Native American women as well. DSM respondents are of younger average age than the population overall. This could be advantageous to the study of high-risk decision making because many risk-management deliberations occur before menopause, but some uses of the data could be helped by recruiting more older respondents. Our sample is also skewed high on both income and education relative to the US population as a whole. This could be a result of collecting data online through databases of research volunteers, who tend to have higher levels of education than the general population [80–82]. Likely impacts of a disproportionally higher-income and more educated sample include underestimates of healthcare access limitations, gaps in our understanding of access barriers, and incomplete ability to understand the complex relationships between education and the understanding and use of risk-related information. Several solutions could facilitate recruiting more members of socioeconomically marginalized populations, who have lower income or education levels, to future datasets: (a) purposefully publicize the survey in socioeconomically marginalized communities and in community-based organizations serving socioeconomically marginalized clients, (b) build community-based research projects that actively partner with community leaders within socioeconomically marginalized communities, (c) identify and recruit high-risk patients from the patient populations of the specific healthcare organizations that largely serve socioeconomically marginalized communities in the US, and (d) collect data by phone or in person within targeted populations.

## Conclusions

The development of the DSM survey and collection of the DSM dataset represent advancements toward the goal of pursuing a more comprehensive understanding of risk-related experiences, coping, and decisions among the full range of women at high risk of breast cancer. The inclusion of both previously validated and newly designed measures covers a wide range of relevant concepts, from personal experiences with the cancers of loved ones and cancer prevention beliefs, to interactions with generalist and specialist healthcare providers, interpersonal support and financial constraints, risk-management decisions and their underlying drivers, and more. This new dataset is largely community-based, opening the door to study the experiences of high-risk women who have not yet accessed clinical care related to that risk. These data also include 35% Black women and 8% women of Ashkenazi Jewish descent, facilitating important analyses of racial and ethnic variation that have been difficult with more homogenous datasets.

## Data Availability

The datasets analyzed for the current study are available from the corresponding author on reasonable request.

## Abbreviations

DSM: Daughter Sister Mother Project—mixed-methods research project on experiences of high-risk women, based at the Ohio State University
FIP: Family Income relative to federal Poverty level for household size
FPL: Federal poverty level
OSU: The Ohio State University
OSUCCC: The Ohio State University Comprehensive Cancer Center
